# Medial Habenula-Interpeduncular Nucleus Circuit Contributes to Anhedonia-Like Behavior in a Rat Model of Depression

**DOI:** 10.3389/fnbeh.2018.00238

**Published:** 2018-10-09

**Authors:** Chunpeng Xu, Yanfei Sun, Xuewei Cai, Tingting You, Hongzhe Zhao, Yang Li, Hua Zhao

**Affiliations:** Department of Physiology, College of Basic Medical Sciences, Jilin University, Changchun, China

**Keywords:** medial habenula, interpeduncular nucleus, chronic unpredictable mild stress, cytochrome c oxidase, depression, substance P

## Abstract

The habenula is a nuclear complex composed of the lateral habenula (LHb) and medial habenula (MHb), two distinct structures. Much progress has been made to emphasize the role of the LHb in the pathogenesis of depression. In contrast, relatively less research has focused on the MHb. However, in recent years, the role of the MHb has begun to gain increasing attention. The MHb connects to the interpeduncular nucleus (IPN) both morphologically and functionally. The MHb-IPN pathway plays an important role in regulating higher brain functions, including cognition, reward, and decision making. It indicates a role of the MHb in the pathogenesis of depression. Thus, we investigated the role of the MHb-IPN pathway in depression. MHb metabolic activity was increased in the chronic unpredictable mild stress (CUMS)-exposed rat model of depression. MHb lesions in the CUMS-exposed rats reversed anhedonia-like behavior, as observed in the sucrose preference test, and significantly downregulated the elevated metabolic activity of the IPN. Substance P (SP)-containing neurons of the MHb were found to innervate the IPN and to be the main source of SP in the IPN. SP content of IPN tissue of the CUMS-exposed rats was increased and MHb lesions reversed this change. In the *in vitro* experiment, firing rate recordings showed that SP perfusion increased the activity of IPN neurons. Our results suggest that hyperactivity of the MHb-IPN circuit is involved in the anhedonia-like behavior of depression, and that SP mediates the effect of the MHb on IPN neurons.

## Introduction

The habenula,located at the dorsal end of the diencephalon, is thought to be a key bridge between the limbic forebrain and midbrain. The habenula forms a nuclear complex that can be further divided into the LHb and MHb. The habenula exerts considerable influence on many behaviors (Lecourtier and Kelly, 2007; Hikosaka et al., 2008). In particular, the LHb has attracted a great deal of interest with respect to its important roles in higher brain functions and the pathogenesis of psychiatric disorders (Geisler and Trimble, 2008; Zhao et al., 2015). Recent evidence has demonstrated that hyperactivity of the LHb is implicated in the pathogenesis of depression (Shumake et al., 2003; Yang et al., 2008). Increased habenular activity may activate GABAergic neurons, which leads to the inhibition of dopaminergic and serotoninergic neurons in the ventral tegmental area (VTA)/substantia nigra (SN) pars compacta and dorsal raphe nucleus (DRN), respectively (Ferraro et al., 1996; Brinschwitz et al., 2010). Considering these results, it is not surprising that the LHb has become the focus of studies in the pathogenesis of depression. However, the other part of the habenula, the MHb, has begun to receive increasing attention in the study of depression. Indeed, MHb dysfunction may also be part of the pathological mechanism for neuropsychiatric illness.

The MHb is different from the LHb both anatomically and histochemically. The MHb is involved in regulating higher brain functions, including reward, attention, mood, and decision making (Lee and Goto, 2011; Fowler et al., 2013; Kobayashi et al., 2013). Abnormality of these functions is accompanied with development of neuropsychiatric disorders (Treadway et al., 2012; Vrieze et al., 2013). Evidence has shown that dysfunction of the MHb contributes to the pathological mechanism of the psychiatric diseases such as attention-deficit/hyperactivity disorder, fear, anxiety, and nicotine addiction and withdrawal (Lecourtier et al., 2004; Lee and Goto, 2011; Kobayashi et al., 2013; Yamaguchi et al., 2013). Congenitally helpless rats have an elevated metabolism in the MHb (Shumake et al., 2003) and tetanus toxin-induced MHb lesions increased fear-induced freezing behavior (Lee et al., 2010). These findings suggest a role of the MHb in the pathogenesis of depression.

The IPN is an important midbrain structure that receives projections mainly from the MHb via the internal portion of the fasciculus retroflexus, and sends fibers to the VTA/SN and DRN, where it regulates the activity of monoaminergic neurons such as DA and 5-HT neurons (Groenewegen et al., 1986; Klemm, 2004; Hikosaka, 2010; Lima et al., 2017). The IPN acts as a bridge between the MHb and monoaminergic system in the regulation of many physiological functions, including reward, learning, sleep, and navigation (McQueen et al., 1984; Morley, 1986; Klemm, 2004; Fowler et al., 2013). Earlier studies showed that increased IPN metabolic activity was correlated with the pathogenesis of depression (Shumake et al., 2003; Padilla et al., 2011). In consideration of the functional relationship between the MHb and the IPN, the influence of the MHb on IPN neuronal activity and, thus, in the pathogenesis of depression, needs to be further investigated.

Studies have shown that hypofunction of acetylcholinergic neurons in the MHb, which project to the IPN, is associated with depression (Han et al., 2017). However, the MHb also releases other neuromodulators, including SP. SP and NK1 receptors are also widely distributed in the cingulate cortex, hippocampus, amygdala, and various hypothalamic areas, which may be associated with the modulation of stress and affective reactions (Nakaya et al., 1994; Ribeiro-da-Silva and Hökfelt, 2000). Stressful stimuli have been shown to induce changes of SP content in some brain areas related to neuropsychiatric disorders (Nakamura et al., 1990; Rosen et al., 1992). Further evidence has revealed that intracerebral injections of SP or NK1 receptor agonists elicit anxiogenic-like effects (Teixeira et al., 1996). Moreover, genetic disruption of the NK1 receptor and injections of NK1 receptor antagonists have been found to generate anxiolytic-like effects in mice and rats, respectively (Santarelli et al., 2001; Rodgers et al., 2004). Selective deletion of the tac1 gene has been reported to diminish anxiety- and depression-related behaviors (Bilkei-Gorzo et al., 2002). Several lines of evidence have also demonstrated that the SP/NK1 receptor system is associated with depression and anxiety (Kormos and Gaszner, 2013). Thus, we conducted the present study to determine whether SP is involved in anhedonia-like behavior evoked by disruption of the MHb-IPN pathway.

## Materials and Methods

### Animals

Male Wistar rats (obtained from the Department of Experimental Animals, Jilin University, Changchun, China) weighing 180–240 g were used for all experiments except for the firing recording experiments of IPN neurons, in which the male Wistar rats used were 90–110 g, 3–4 weeks of age. Rats were housed in a standard cage, with free access to food and water, and in a temperature-controlled room (22 ± 2°C, 12-h light–dark cycle, lights on at 7 a.m.). All procedures were conducted in accordance with the Guide for the Care and Use of Laboratory Animals and were approved by the Jilin University Animal Care Research Committee.

### Chronic Unpredictable Mild Stress-Induced Model of Depression

Rats were subjected to a series of unpredictable and mild stressors, as previously described (Roth and Katz, 1981) but with slight modifications. Briefly, two different stressors per day were randomly selected from eight possible stressors and applied for 28 days. The stressors were as follows: a 45° cage tilt for 24 h; food deprivation for 40 h; swimming in cold water at 4°C for 5 min; water deprivation for 24 h; a thermal environment at 45°C for 5 min; reversal of the light/dark cycle; tail clips for 1 min; and wet bedding for 24 h. Control animals were housed in a separate room and were not subjected to any stressors.

### Behavioral Verification of the Chronic Unpredictable Mild Stress Model

#### Sucrose Preference Test

Anhedonia induced by a CUMS protocol was assessed using the SPT. Rats were habituated to a 1% sucrose solution (w/v) prior to the test. Two bottles of sucrose solution were placed in each cage for 72 h and the 1% sucrose solution was replaced by water for the subsequent 24 h. After 20 h of food and water deprivation, sucrose and water consumption were measured within a 24 h testing window. The sucrose preference was calculated using the following formula: percentage sucrose preference = (sucrose solution consumption/total liquid intake) × 100.

#### Forced Swimming Test

The FST was conducted as previously described (Yang et al., 2008). Rats were placed in a cylinder with a 20 cm internal diameter × 50 cm height that was filled with water at 25 ± 1°C and without the possibility of escape. Rats were forced to swim for 15 min; 24 h after this first exposure, a test trial was carried out for 5 min. After each session, the cylinder was cleaned and refilled with fresh water. Immobility time was considered as a measure of behavioral despair, and was recorded by investigators who were blind to the experiment.

### MHb Lesions

The CUMS-exposed rats were anesthetized with 10% chloral hydrate (i.p., 0.35 g/kg) and immobilized with a stereotaxic instrument (Narishige Corporation, Japan). Then, an electrode with a 35–45 kΩ resistance was inserted bilaterally into the MHb (3.3–3.5 mm posterior to bregma, 1.5–1.6 mm lateral to the midline, and 4.2–4.5 mm ventral to the surface of the skull) at a 15° angle. A 0.30 mA DC current was administered for 40 s using an electronic stimulator (SEN-7130, Nihon Kohden Kogyo Co., Ltd., Tokyo, Japan). Rats in the sham-lesion group received the same treatment but without a current. All animals were allowed 7 days of recovery before behavioral or neurochemical tests. The position of the MHb lesions was identified histologically (**Supplementary Figure S1**).

### IPN Neuronal Firing Rate Recording

#### IPN Slice Preparation

Rats were anesthetized with 20% urethane (i.p., 1.2 g/kg). When deeply anesthetized, the brain was excised and suspended immediately in ice-cold ACSF with 95% O_2_ and 5% CO_2_ for brain slice preparation. The tissue was trimmed to the brainstem region, and 400-μm thick coronal brain slices containing the IPN were cut using a vibratome (5000 mz^-2^, Campden Instruments Ltd., United Kingdom). Slices were incubated in ACSF at 34°C for 25 min and then allowed to equilibrate in the ACSF at 22–25°C for at least 1 h before the recordings. Slices were then transferred to the recording chamber and infused with oxygenated ACSF (22–25°C) at a flow rate of 2 ml/min.

#### Firing Recording and Substance P Administration

The firing rate of IPN neurons was recorded using a glass microelectrode filled with a 2% solution of pontamine sky blue in 0.5 mol/l sodium acetate buffer. Electrode impedance ranged from 13 to 15 MΩ. Extracellular potentials were amplified and filtered with a microelectrode amplifier (FWD-1A, Liuhe Radio Factory, Nanjing, China). Action potentials were monitored continuously using a dual-beam storage oscilloscope (VC-10, Nihon Kohden, Tokyo, Japan) and collected and digitized using a data acquisition system with a sampling rate of 10 kHz (PowerLab 4/25; ADI, Sydney, Australia). SP (ab120170, Abcam, United Kingdom) was dissolved in sterile distilled water, diluted in ACSF, and then perfused into the slice chamber at a concentration of 10 nM in 20 ml. The reference point for classification of neurons is 20%. It means that when the firing rate increased by 20% or more, we considered it as increased or enhanced. If the firing rate decreased by 20% or more, we considered it as reduced or declined. When firing rate changed less than 20%, it was considered as unchanged. The recording positions were confirmed by ejecting pontamine shy blue after the completion of recording.

### Measurement of Cytochrome c Oxidase Activity

Rats were injected with an overdose of chloral hydrate. Using the vibration cutting machine (ZQP-86, Shanghai five phase instrument Co., Ltd., Shanghai, China), we collected two 500-μm-thick brain slices containing the MHb from coronal sections between Bregma -3.1 and -4.1 mm, where the MHb take larger area than in other locations. Then we transferred the slices into a glass dish with ice-cold ACSF. Under the microscope, the MHb was cut off from the inner side close to the boundary between the MHb and LHb with a 25-g thin needle. The tissue was washed with 0.1 M ice-cold PBS and ground with a glass homogenizer (1 ml) on ice. Isolation of mitochondria was performed using the Tissue Mitochondria Isolation Kit (Beyotime Inst. Biotech, Peking, China). The homogenates were centrifuged at 600 × *g* at 4°C for 5 min, and the pellet was discarded. Next, the supernatant was centrifuged at 11,000 ×*g* for 10 min, and the new supernatant was discarded. Then, the pellet was resuspended in enzyme dilution buffer. CCO activity was analyzed using a CCO Assay Kit (CYTOCOX1, Sigma, St. Louis, MO, United States), according to the manufacturer’s instructions. CCO activity was measured in a spectrophotometer (Ultrospec3100pro, Amersha, Sweden) as the decrease in OD_550nm_ absorbance of ferricytochrome c due to its oxidation by CCO. One unit of CCO activity was defined as the oxidization of 1.0 mol of ferricytochrome c per minute at pH 7.0 and 25°C.

### Substance P Measurement

Interpeduncular nucleus tissue punches were processed immediately after collection. IPN tissues were weighed and homogenized in 2 M ice-cold acetic acid (v/w = 75 μl/1 mg) and then punched with disposable pellet pestles in microtubes (1.5 ml) on ice. Homogenates were centrifuged at 16,100 *g* and 4°C for 10 min. The supernatants were carefully removed and the pH was neutralized with 1 M sodium hydroxide. SP concentrations were determined using an EIA kit (item no 583751, Cayman Chemicals, Ann Arbor, MI, United States) according to the manufacturer’s instructions. All samples were diluted to yield SP concentrations within the linear range of the standard curve (3.9–500 pg/ml). SP content was expressed as pg/mg wet tissue weight.

### Statistical Analysis

Normality of the group samples was assessed using the Kolmogorov–Smirnov and Shapiro–Wilk normality tests. Equality of variances was assessed using Levene’s test. When normality and equal variance between sample groups was achieved, *t*-tests were used for between-group comparisons. When data were non-normal or equality of variance of samples failed, Mann–Whitney U or Wilcoxon signed rank tests were performed. All statistical tests were two-tailed. Significance was set at *P* < 0.05. SPSS v23.0 and GraphPad Prism v7.0 were used for statistical analysis and generation of graphics.

## Results

### Behavioral Validation of the Chronic Unpredictable Mild Stress Model

**FIGURE 1:**
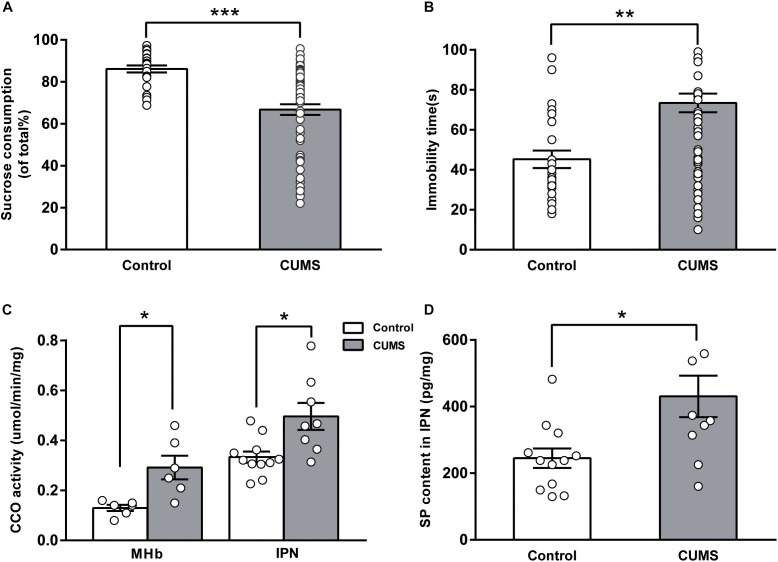
Changes in cytochrome c oxidase activity and SP content of the MHb-IPN pathway in rats exposed to chronic unpredictable mild stress. Validation of depression-like behaviors: results of the sucrose preference test **(A)** and the forced swimming test **(B)** in CUMS-exposed rats are shown. Increased cytochrome c oxidase activity **(C)** of the MHb and the IPN, and substance P content of the IPN **(D)** in CUMS-exposed rats are shown. Statistical analysis was performed using Mann–Whitney *U*-tests **(A,B)** and Student’s *t*-tests **(C,D)**. Data are presented as the mean ± SEM. ^∗^*p* < 0.05, ^∗∗^*p* < 0.01, ^∗∗∗^*p* < 0.001 compared with the control rats.

The CUMS-exposed rats (*n* = 68) exhibited a significantly lower sucrose consumption in the SPT than in the control rats [*n* = 24, percentage sucrose preference in the CUMS-exposed rats: 66.75 ± 2.57%, 95% confidence interval [CI] (61.62, 71.88); control rats: 86.14 ± 1.70%, 95% CI (82.63, 89.65); *U* = 311, *P* < 0.001, **Figure 1A**]. Compared with control rats, the CUMS-exposed rats had a markedly longer immobility time in the FST [CUMS-exposed rats: 73.46 ± 4.69 s, 95% CI (64.09, 82.82); control rats: 45.25 ± 4.40 s, 95% CI (36.16, 54.34); *U* = 447, *P* = 0.001, **Figure 1B**]. These results indicate that the CUMS-exposed rats developed a state of anhedonia and behavioral despair.

### Increased CCO Activity in the MHb-IPN Pathway of CUMS-Exposed Rats

Differences in CCO activity of the MHb-IPN pathway were compared between the control rats and the CUMS-exposed rats. CCO activity of the MHb in the CUMS-exposed rats [0.29 ± 0.05 μmol/min/mg, 95% CI (0.17, 0.41), *n* = 6] was significantly greater than that of the control rats [0.13 ± 0.01 μmol/min/mg, 95% CI (0.10, 0.16), *n* = 6; *t* = -3.326, *p* = 0.017, **Figure 1C**]. CCO activity of the IPN in the CUMS-exposed rats [0.50 ± 0.05 μmol/min/mg, 95% CI (0.37, 0.62), *n* = 8] was significantly greater than that of the control rats [0.33 ± 0.02 μmol/min/mg, 95% CI (0.28, 0.38), *n* = 11; *t* = -2.788, *p* = 0.02, **Figure 1C**]. These results indicate that neuronal activity of the MHb-IPN pathway was significantly increased in the CUMS-exposed rats.

### Elevated SP Content in the IPN of CUMS-Exposed Rats

Substance P in the brain modulates physiological and behavioral stress responses and affective behaviors. In the enzyme-linked immunosorbent assay analysis, we found that SP concentration in the IPN was significantly elevated in the CUMS-exposed rats [430.58 ± 62.12 pg/mg, 95% CI (290.1, 571.1), *n* = 10] compared with that of the control rats [245.16 ± 29.34 pg/mg, 95% CI (180.6, 309.7), *n* = 12; *t* = -2.699, *p* = 0.018, **Figure 1D**]. Tissue content of neurokinins has been widely used to estimate the release of neurokinins after challenges such as aversive and stressful experiences (Herpfer and Lieb, 2005). Our results indicated that the release of SP in IPN was increased in the CUMS-exposed rats.

### Effects of MHb Lesions on Depression-Like Behavior in CUMS-Exposed Rats

**FIGURE 2:**
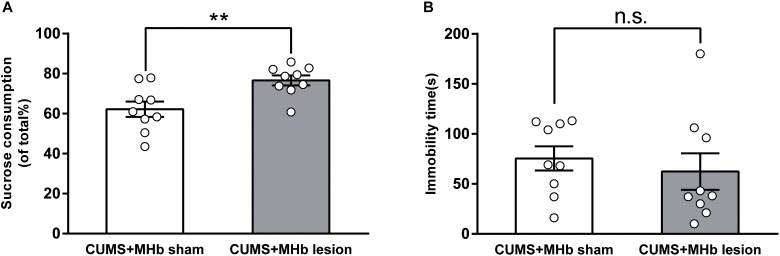
Behavioral changes of CUMS-exposed rats after MHb lesions. MHb lesions (but not sham lesions) reversed the preferences in the sucrose preference test **(A)**. The forced swimming test results **(B)** were not significantly different between the two groups. Statistical analysis was performed using Student’s *t*-tests **(A)** and Mann–Whitney *U*-tests **(B)**. Data are presented as the mean ± SEM. ^∗∗^*p* < 0.01, n.s., not significant compared with the control rats.

A total of 24 CUMS-exposed rats were used for the MHb lesion experiment. These rats were divided into CUMS + MHb-lesion and CUMS + MHb-sham rats. Rats identifiable with the MHb lesions were included in the statistical analysis. Behavioral tests were performed 1 week after the MHb lesions. The MHb-lesion rats consumed significantly more sucrose than the MHb-sham rats in the SPT [percentage sucrose preference in CUMS + MHb-lesion rats, 76.64 ± 2.50%, 95% CI (70.86, 82.41), *n* = 9; CUMS + MHb-sham rats, 62.19 ± 3.82%, 95% CI (53.39, 70.99), *n* = 9; *t* = 3.166, *P* = 0.006, **Figure 2A**], which suggested a difference in their hedonic state. No significant between-group difference in immobility time was detected in the FST [CUMS + MHb-lesion rats, 62.33 ± 18.25 s, 95% CI (20.24, 104.4), *n* = 9; CUMS + MHb-sham rats, 75.44 ± 12.08 s, 95% CI (47.58, 103.3), *n* = 9; *U* = 27.5, *p* = 0.258, **Figure 2B**]. Our results indicated that the MHb lesions had a marked effect on hedonic state, but not on behavioral despair. The most straightforward explanation for the discordant results is that the MHb plays a role in mediating motor activity.

### Decreased CCO Activity and SP Content in the IPN After MHb Lesions in CUMS-Exposed Rats

**FIGURE 3:**
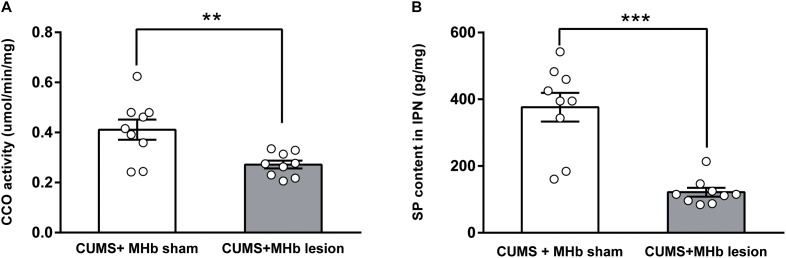
Cytochrome c oxidase activity and substance P content of the IPN after MHb lesions in CUMS-exposed rats. MHb lesions decreased cytochrome c oxidase activity **(A)** and substance P content **(B)** in the IPN. Statistical analysis was performed using Student’s *t*-tests **(A)** and Mann–Whitney *U*-tests **(B)**. Data are presented as the mean ± SEM. ^∗∗^*p* < 0.01, ^∗∗∗^*p* < 0.001 compared with the control rats.

The MHb innervates multiple subnuclei of the IPN via the fasciculus retroflexus. To determine whether MHb lesions altered the functional neuronal activity of the IPN, we examined the differences in the CCO activity of the IPN between the CUMS + MHb-lesion and CUMS + MHb-sham rats. The CUMS + MHb-lesion rats had less CCO activity in the IPN than the CUMS + MHb-sham rats [CUMS + MHb-lesion rats, 0.27 ± 0.02 μmol/min/mg, 95% CI (0.24, 0.31), *n* = 9; CUMS + MHb-sham rats, 0.41 ± 0.04 μmol/min/mg, 95% CI (0.32, 0.50), *n* = 9; *t* = 3.219, *p* = 0.005, **Figure 3A**]. Our results suggest that MHb lesions induced a functional decline in IPN neuronal activity in CUMS-exposed rats.

To verify the effects of MHb lesions on SP release, we compared the concentration of SP in the IPN between the two experimental groups. MHb lesions in CUMS-exposed rats markedly decreased SP release to the IPN, as the concentration of SP in the IPN was significantly decreased compared with that of the MHb-sham rats [CUMS + MHb-lesion rats, 121.58 ± 13.23 pg/mg, 95% CI (91.07, 152.1), *n* = 9; CUMS + MHb-sham rats, 376.41 ± 43.06 pg/mg, 95% CI (277.1, 475.7), *n* = 9; *U* = 2.0, *p* < 0.001, **Figure 3B**].

### Excitatory Effects of SP on the Firing Rate of IPN Neurons

**FIGURE 4:**
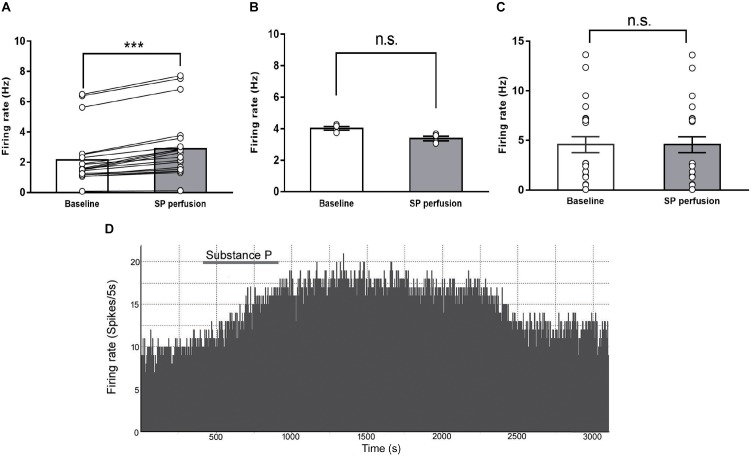
The effects of substance P on the firing rates of IPN neurons in rat brain slices. The spontaneous firing rates of IPN neurons in response to substance P perfusion increased (**A**, *n* = 20), declined (**B**, *n* = 4), or were unchanged (**C**, *n* = 24). A representative of the spontaneous firing of IPN neurons activated after substance P perfusion **(D)**. Statistical analysis was performed with Wilcoxon signed rank test. Data are presented as mean ± SEM. ^∗∗∗^*p* < 0.001, n.s., not significant compared with the control group.

Substance P can change the neuronal activity in several brain regions (Baulmann et al., 1999; Spitznagel et al., 2001). To test the hypothesis that SP release increases the neuronal activity of the IPN, we recorded firing rate extracellularly from IPN neurons using an *in vitro* slice preparation and validated the pharmacological signature of IPN neurons responding to SP perfusion. A total of 48 IPN neurons were recorded from slices obtained from 42 rats; among them, 20 neurons showed enhanced firing rates. The spontaneous activity after SP perfusion increased by an average of 40.8 ± 5% [from 2.15 ± 0.41 Hz, 95% CI (1.29, 3.02) to 2.88 ± 0.48 Hz, 95% CI (1.87, 3.89), *P* < 0.001, **Figure 4A**]. The latency to the onset of activation was 154.3 ± 10.8 s (range: 81–223 s), and the average duration of activation was 2071 ± 341 s (range: 382–4673 s). A representative of the spontaneous firing of IPN neurons activated after substance P perfusion was showed in **Figure 4D**. Meanwhile, among the 48 neurons tested, four were inhibited and 24 neurons showed unresponse to SP perfusion, these results are shown in **Figures 4B,C** respectively. The SP induces three different responses in the IPN, possibly because the SP acts on different neurons, which express SP receptors. All the recorded neurons in the IPN, including excitatory, inhibitory, and unresponsive neurons, as a whole, showed small but significant differences between baseline and post-SP perfusion [3.52 ± 0.46 Hz, 95% CI (2.59, 4.45) versus 3.76 ± 0.46 Hz, 95% CI (2.84, 4.68); *p* = 0.01]. These results reveal an excitatory effect of SP on the firing rate of IPN neurons.

## Discussion

Some studies have shown that the MHb has a considerable influence on many behaviors, including stress responses, reward, misery-fleeing, and cognition. Disruptions of these behaviors may be a pathological mechanism underlying neuropsychiatric disorders (Agetsuma et al., 2010; Lee et al., 2010; Viswanath et al., 2013). In the present study, we used the CUMS paradigm in rats to create a model of depression, which has been validated in many laboratories for the studies of the pathogenesis of depression (Willner, 2017). After 28 days of CUMS treatment, rats developed depression-like behaviors. We found that CUMS-exposed rats consumed less sucrose in the SPT and were immobile for longer in the FST. CCO activity of the MHb, which is closely correlated with neuronal functional activity (Wong-Riley, 1989; Wong-Riley et al., 1998), was significantly higher in CUMS-exposed rats than that in the control rats. The decreased sucrose preference of CUMS-exposed rats, which is known as an anhedonia-like behavior, was reversed by MHb lesions. The detectable elevation in CCO activity of the MHb was consistent with observations reported by Shumake et al. (2003) in genetically helpless rats. These results are also in agreement with those reported by Febbraro et al. (2017), who showed that the expression of c-fos was significantly increased in the MHb of CUMS-exposed rats. Altogether, these results suggest that increased metabolism of the MHb may be associated with depression.

The increase of immobility time in CUMS-exposed rats, an indicator of behavioral despair, was not reversed after MHb lesions. Consistent with this finding, mice with genetically targeted MHb lesions showed no deficits in the FST in a previous study (Hsu et al., 2014). Another study found that reduced expression of choline acetyltransferase in rats to selectively suppress the MHb cholinergic circuit had no effect on immobility time in the FST (Han et al., 2017). The factors that influence motor activity can also affect immobile behavior in the FST. For example, motor-impairing compounds can lead to false-negative results in the FST (Slattery and Cryan, 2012). Several studies have demonstrated that the MHb may affect motor activity. The recordings of MHb neuronal firing in rats during a pellet-chasing task found that cellular activity in a reasonably large portion of the MHb was significantly associated with running speed, whereby faster running was accompanied by higher firing rates in MHb neurons (Sharp et al., 2006). Mice with genetically targeted MHb lesions have been found to exhibit a shorter latency to fall compared with control mice in the rotarod test, which indicates a deficit in the ability or drive of the animal to maintain an upright position (Hsu et al., 2014). Thus, the effects of MHb lesions on motor activity and/or decision making during forced swimming stress may interfere with MHb lesion-induced improvements in immobility time in CUMS-exposed rats. Alternatively, the MHb is involved in the stress response, which may be related to its release of SP (Yamaguchi et al., 2013). SP regulates HPA axis activity under stress conditions (Ebner and Singewald, 2006), which has been proposed to underlie the increased immobility in the FST (Schmidt et al., 2009). MHb lesions may induce an inhibition of the SP system, which in turn activates the HPA axis through a negative feedback mechanism, leading to an increased immobility time.

The MHb sends dense efferent projections to the mesencephalic IPN, and regulates the activity of IPN neurons (Herkenham and Nauta, 1977; Lima et al., 2017). We found that CCO activity in the IPN was significantly increased in CUMS-exposed rats. Earlier research has found that congenitally helpless rats had a 25% elevation in metabolism in the IPN compared with a control group (Shumake et al., 2003). Using the same congenitally helpless rats, Padilla et al. (2011) found that a protective effect of fluoxetine correlated with changes in regional CCO activity in a variety of brain networks, including the IPN; this indicates that increased IPN metabolic activity was correlated with depression. Our investigation showed that MHb lesions suppressed CCO activity of the IPN in CUMS-exposed rats, which suggests that the alleviation of anhedonia-like behavior in CUMS-exposed rats may be related to the decreased activity of IPN neurons. Collectively, the MHb is connected to the IPN both morphologically and functionally. The functions of the MHb related to sleep, reward, and motor activity require the involvement of the IPN (Klemm, 2004). Together with the present results, these findings indicate that the effect of MHb lesions on ameliorating anhedonia-like behavior in CUMS-exposed rats may be mediated by altering the activity of the IPN neurons.

A large number of SP neurons are distributed in the MHb. SP has been implicated in the regulation of many behavioral and cognitive functions, including depression-related behaviors. In rodents, for example, an increased SP concentration was found in the septum and dentate gyrus after foot shock (Siegel et al., 1984), and in the periaqueductal gray after restraint stress (Rosen et al., 1992). In humans, serum SP levels were found to be elevated in patients suffering from major depression. Moreover, antidepressant treatment has been found to reduce SP serum levels (Bondy et al., 2003; Lieb et al., 2004), and clinical trials using NK1 antagonists have revealed robust antidepressant effects (Kramer et al., 1998). These studies support the idea that SP has a close connection with depression. SP neurons in the MHb project to the IPN. Thus, we investigated whether SP in the IPN can mediate the effect of MHb lesions on depression-like behavior in CUMS-exposed rats. We found that SP content of the IPN was elevated in stressed rats compared with that of the control rats. These results were consistent with the increased CCO activity of the IPN in CUMS-exposed rats. Furthermore, MHb lesions in CUMS-exposed rats recovered sucrose consumption and decreased SP content in the IPN compared with that in the MHb-sham lesion rats. Therefore, changes in the SP content of the IPN may mediate the behavioral improvement in SPT after MHb lesions. To determine how SP impacts IPN neurons, we tested the effect of SP perfusion on the firing rates of IPN neurons by extracellularly recording *in vitro* slice preparations. The results showed that the spontaneous activity of 42% of IPN neurons was enhanced after SP perfusion, which is suggestive of an excitatory effect of SP on IPN neuronal activity. Together, these results suggest that the hyperactivity of MHb in CUMS-exposed rats may cause an elevated activity of the IPN neurons by increasing the release of SP from the MHb to the IPN. The behavioral reversal found in the MHb lesion rats also supports this idea. In addition, a large number of cholinergic neurons are distributed in the MHb. The disorder of cholinergic neurons in the MHb contributes to the anhedonia-like behavior of chronic restraint stress-exposed rats (Han et al., 2017). However, in chronic restraint stress-exposed rats, a model of depression, the expression of cholinergic signaling genes in the MHb was downregulated. Obviously, the improvement in anhedonia-like behaviors of CUMS-exposed rats by MHb lesions is irrelevant to the inhibition of cholinergic signaling.

## Conclusion

In our study, CUMS exposure in rats generated a valid model of depression. In this model, we found elevated neuronal activity of the MHb-IPN pathway, which was coupled with the elevation of SP content in the IPN. The CUMS-exposed rats with MHb lesions exhibited a reversal of anhedonia-like behavior. In addition, we found that CCO activity and SP content were downregulated in the IPN, which was accompanied by the recovery of anhedonia-like behavior. Finally, firing recordings of IPN neurons in brain slices showed that the spontaneous activity of IPN neurons was significantly increased in response to SP perfusion. Collectively, our results suggest that, in CUMS-exposed rats, an increase in the neuronal activity of the MHb-IPN circuit was involved in the performance of anhedonia-like behavior and that SP might mediate the effect of the MHb on IPN neurons.

## Author Contributions

HuZ and CX designed the experiments. CX, YS, XC, TY, and HoZ executed the experiments. YS, YL, and CX analyzed the data. CX, YL, and HuZ drafted the manuscript and prepared the figures. All authors approved the final version of the manuscript.

## Conflict of Interest Statement

The authors declare that the research was conducted in the absence of any commercial or financial relationships that could be construed as a potential conflict of interest.

## Supplementary Material

FIGURE S1 A white filed imaging of MHb lesion. Scalebar = 200 μm. MHb, medial habenula; LHb, lateral habenula; PVP, posterior paraven-tricular nucleus of the thalamus; GrDG, granular layer dentate gyrus; D3V, dorsal third ventricle.

## Abbreviations

ACSF: artificial cerebrospinal fluid
CCO: cytochrome c oxidase
CUMS: chronic unpredictable mild stress
FST: forced-swim test
HPA: hypothalamic-pituitary-adrenal
IPN: interpeduncular nucleus
LHb: lateral habenula
MHb: medial habenula
NK1: neurokinin 1
SP: substance P
SPT: sucrose preference test
