# Supplementary figures and images for: Medial Habenula-Interpeduncular Nucleus Circuit Contributes to Anhedonia-Like Behavior in a Rat Model of Depression
Source: Front Behav Neurosci. 2018 Oct 9;12:238. doi: 10.3389/fnbeh.2018.00238 (PMC6189744; doi:10.3389/fnbeh.2018.00238)

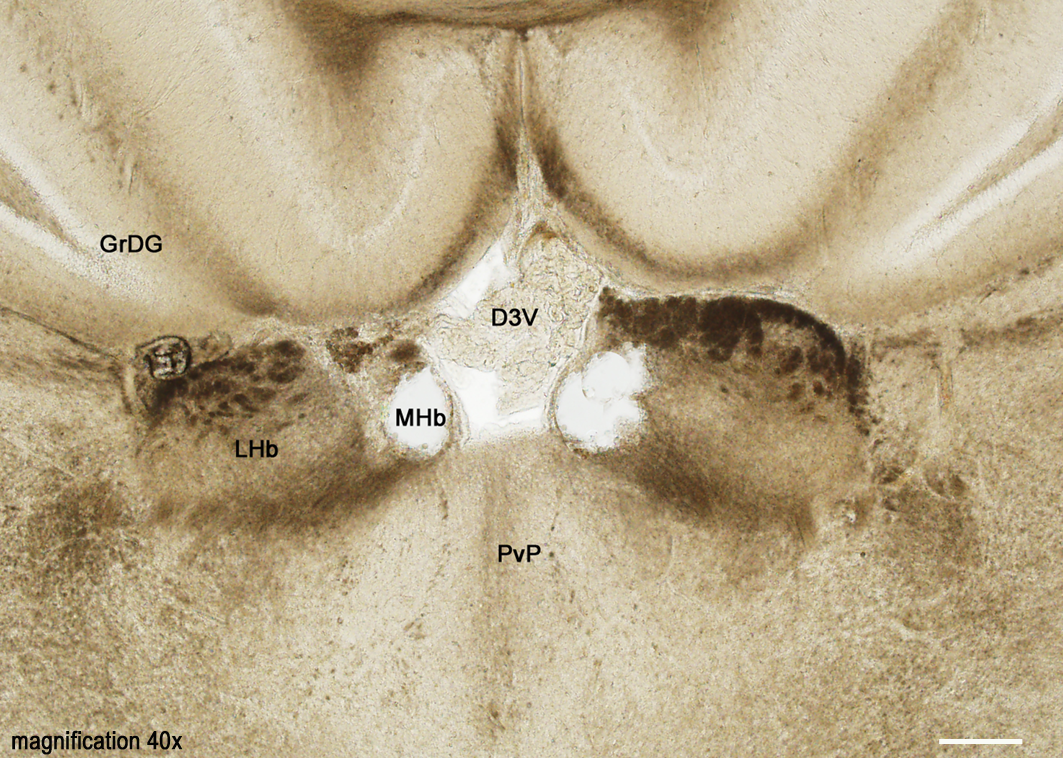

Supplement: FIGURE S1 — A white filed imaging of MHb lesion. Scalebar = 200 μm. MHb, medial habenula; LHb, lateral habenula; PVP, posterior paraven-tricular nucleus of the thalamus; GrDG, granular layer dentate gyrus; D3V, dorsal third ventricle. [file Image_1.TIF]
